# Examining the Impact of Group-Based Social Skills Intervention in Autistic Children Aged Eight to 15 Years

**DOI:** 10.7759/cureus.53376

**Published:** 2024-02-01

**Authors:** Mona P Gajre, Mansi Shah, Shreeya V Pradhan, Harshna Aseri

**Affiliations:** 1 Department of Pediatrics, Lokmanya Tilak Municipal Medical College and General Hospital, Mumbai, IND

**Keywords:** adolescence, social skills therapy, social communication, group intervention, behavior challenges, autism spectrum disorder (asd)

## Abstract

Introduction

Autism spectrum disorder (ASD) is a neurological and developmental disorder, which poses challenges to social communication and behavior, particularly affecting social functioning. Individuals with ASD face significant social challenges, including difficulty understanding social cues and body language, limited ability to engage in reciprocal social interactions, and challenges with establishing empathy. A preference for routines and repetitive behaviors limits their ability to adapt to new or unexpected social situations. These problems tend to escalate during adolescence. These often cause distress to the individual as well as the caregivers. Group-based social skills interventions (GSSIs) are a widely used and effective modality for addressing core social impairments in children with autism. This study aims to assess the impact of GSSI on the broad age group of eight to 15 years, involving parents to enhance the transferability of children's social skills.

Methods

This was a single-arm interventional study where 30 verbal autistic children, aged eight to 15 years, with intelligence quotient (IQ) > 70 were enrolled after utilizing the Binet Kamat Test of Intelligence (BKT) to assess IQ and the Indian Scale for Assessment of Autism (ISAA) to grade severity of autism. The children received GSSI from interdisciplinary therapists for 12 sessions, on a weekly basis, lasting 90 minutes each for a period of three months. After each therapy session, parents received summaries of each session and were delegated reinforcing homework assignments to enable generalization and maintenance of the skills taught. Outcome measures were taken at three points in time by utilizing the Social Communication Questionnaire (SCQ) and the parent-rated Social Responsiveness Scale 2 (p-SRS-2): T1: pre-therapy at the time of enrolment; T2: immediately post-therapy at the end of three months of training; and T3: long-term follow-up, three months after the end of training.

Results

Mean SCQ scores were as follows: T1 = 21.87, T2 = 18.57, and T3 = 18.57 (p = 0.000). This progressive decline at T1, T2, and T3 indicated a decreasing trend in the severity of difficulties in the social communication domain. Mean p-SRS-2 scores were as follows: T1 = 73.00, T2 = 64.57, and T3 = 64.30 (p < 0.0001). This declining trend at T1, T2, and T3 suggested a statistically significant decrease in the severity of difficulties faced in various social aspects tested by the p-SRS-2, i.e., social awareness, social cognition, social communication, and social motivation, along with a reduction in restricted interests and repetitive behaviors (RRBs). Very strong correlation coefficients were obtained for SCQ scores (T1-T2 = 0.921, T1-T3 = 0.921, and T2-T3 = 1.000), as well as for p-SRS-2 scores (T1-T2 = 0.743, T1-T3 = 0.746, and T2-T3 = 0.989), which reinforced the statistical significance of the data.

Conclusion

GSSI is an effective parent-assisted intervention for adolescents with ASD, with effects lasting up to three months post-intervention.

## Introduction

Autism spectrum disorder (ASD) is a developmental disorder that impacts communication and behavior [1]. Global estimates suggest that approximately one in 100 children is affected by autism, with this figure serving as an average. However, reported prevalence rates exhibit considerable variation across different studies [2]. While it can be diagnosed at any age, it is referred to as a "developmental disorder" as symptoms typically manifest within the first two years of life. Deficiency in social-emotional reciprocity, i.e., the inability to effectively engage with others and share thoughts and feelings, is evident early in children with ASD. The challenges that have the most profound and defining impact on individuals with ASD are the ones linked to impaired social functioning [3]. Difficulty with verbal as well as non-verbal forms of communication can lead to major challenges in the expression of thoughts, feelings, and needs, and misinterpretation of others’ intentions and emotions can lead to misunderstandings and conflicts. Heightened sensitivities to sensory stimuli can make social environments overwhelming, leading to avoidance of ordinary social situations [4]. Children with ASD experience a heightened difficulty in social communication during adolescence, necessitating an enhanced understanding of peer interactions [5].

Socialization groups are a widely used modality for addressing core social impairments in verbal, school-aged, and older individuals with ASD. They hold appeal as a cost-effective method to facilitate social contact for those at increased risk for social isolation and rejection [6]. A recent study by Gajre et al. showed a reassuring improvement in social skills after the implementation of social skills group therapy in the age group of eight to 12 years [7]. Our study was conducted with the aim to extend the findings to the larger age group of eight to 15 years using group-based social skills intervention (GSSI) and to enhance the transferability of children's social skills by involving parents and/or teachers in the same, as they can actively assist the children in applying the training in their everyday routines.

## Materials and methods

This was a single-arm interventional study conducted at the Autism Intervention Center affiliated with Lokmanya Tilak Municipal General Hospital, Mumbai, India, over a time period of six months. The study was commenced after ethical clearance was granted by the Institutional Review Board (Ref No.: D02018014, IEC 283/18; dated: 24.01.2018).

The study involved 30 verbal children diagnosed with ASD, aged eight to 15 years, whose parents consented to their participation, with intelligence quotient (IQ) > 70, who were enrolled and assessed prior to targeted interventions (T1). The inclusion criteria involved children who met the Diagnostic and Statistical Manual of Mental Disorders, 5th Edition criteria for diagnosis of autism, with a verbal IQ score > 70, and those who were able to follow instructions by therapists. Children without any major physical condition who could travel to the training center for the sessions without any difficulty were preferred. The exclusion criteria involved children who had started a new psychiatric medication within 30 days prior to enrolment, those with recognized moderate to severe intellectual disabilities or an ongoing seizure disorder, individuals displaying self-injurious behaviors or aggression toward others, and those with any psychiatric comorbidities or evident genetic syndromes.

**Figure 1:**
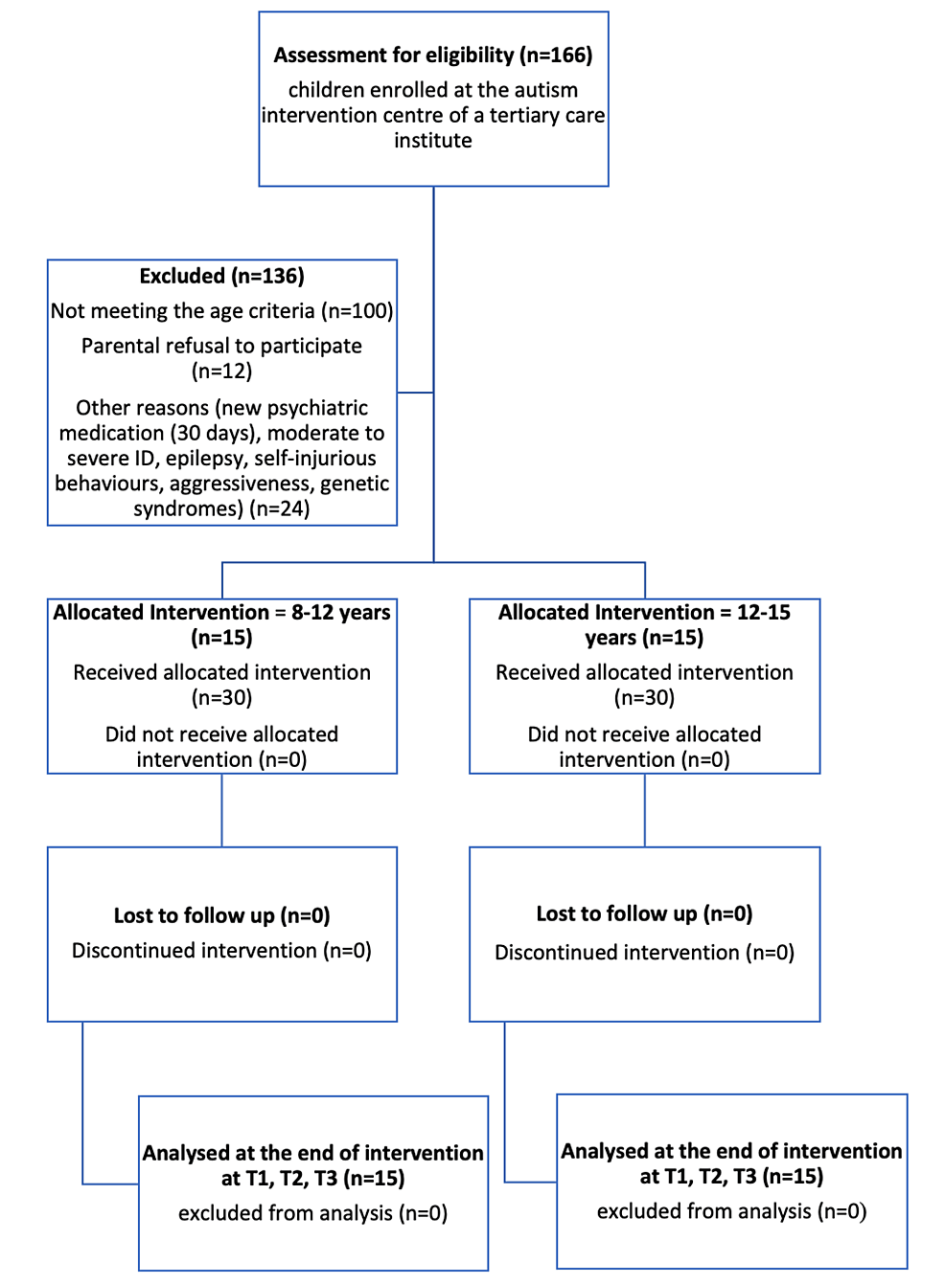
Flow diagram depicting single-arm study design

The enrolled children were divided into two age groups, i.e., eight to 11 years and 12-15 years, with a maximum of 15 children assigned to each therapy group (Figure 1).

Once enrolled, the children received GSSI from interdisciplinary therapists (speech-language pathologists and occupational therapists) with a child-to-therapist ratio of 3:1. Both groups of children underwent an identical training regimen, which comprised 12 sessions lasting 90 minutes each. These sessions were conducted on a weekly basis, spanning a period of three months. Due to the variation in social situations experienced by the age groups of eight to 11 years and 12-15 years, distinct lesson plans were developed for each group, as shown in Table 1.

**Table 1:** Weekly lesson plans for group-based social skills intervention

Week	Group A: 8-11 years	Group B: 12-15 years
1	Introduction and trading information	Introduction and trading information
2	Two-way conversations	Two-way conversations
3	Starting and joining conversations	Starting and joining conversations
4	Exiting conversations	Exiting conversations
5	Choosing appropriate friends	Choosing appropriate friends
6	Teamwork	Good sportsmanship
7	Handling physical bullying	Handling arguments
8	Problem-solving	Appropriate use of humor
9	Competitive spirit	Changing reputations
10	Get-togethers	Handling teasing and embarrassing feedback
11	Good sportsmanship	Handling physical bullying
12	Final review, post-test assessment, and graduation	Final review, post-test assessment, and graduation

Each session followed a pre-defined structure, which included 15 minutes of free play to help the children feel comfortable, 60 minutes of group training, and 15 minutes of circle time, where the learning of the day would be summarized. Children received group training through instruction, directed positive feedback, observation, and roleplay. The therapists simultaneously analyzed the behavior of the children, defined individual positive target behaviors, such as appropriate social etiquettes, body language, and other verbal and non-verbal means of communication, and elicited positive behavior by demonstrations. Negative behavior was ignored when possible, while differentially reinforcing alternative or compatible positive behavior. Alongside the group training, parents were given a summary of the skills taught during each session. They were explained the goal of the training session, and instructed regarding the application of these skills in home and community settings. After the therapy sessions concluded, parents were tasked with ongoing assignments to be completed at home. These assignments were curated with the aim of reinforcing the skills taught in the respective training session, thus enabling generalization and maintenance of the skill. For instance, following the session on "Introduction and trading information," parents were tasked with encouraging the child to reciprocate the same at home in a telephonic conversation with another peer. Other assignments involved identifying a suitable extracurricular activity for the child and enrolling them in it. Nevertheless, the primary focus of these assignments was to practice the skills imparted during the sessions. Assessments were conducted before the intervention (T1), at the end of intervention (T2), and three months after the end of intervention (T3).

Tools used for assessment

Before the initiation of GSSI (at T1), the Binet Kamat Test of Intelligence (BKT) was utilized to assess the IQ of children by a trained psychiatrist [8], and the Indian Scale for Assessment of Autism (ISAA) was employed to grade the severity of autism [9]. It consists of 40 items divided into six domains: social relationship and reciprocity, emotional responsiveness, speech-language and communication, behavior patterns, sensory aspects, and cognitive components. The scoring process, conducted by investigators, involved reviewing information provided by parents and observing the child, which typically took 20-30 minutes to complete.

To assess the outcomes before (T1), immediately after (T2), and three months after (T3) intervention, two parent-rated questionnaires, i.e., the Social Communication Questionnaire (SCQ) and the Social Responsiveness Scale 2 (p-SRS-2), were utilized. The SCQ was originally designed as a questionnaire version of the Autism Diagnostic Interview-Revised (ADI-R). Multiple studies have established it as a reliable tool for screening ASD and for understanding the individual's current daily experiences, thus supporting the evaluation of therapeutic approaches [10]. It was completed by the primary caregiver, who is most involved in the day-to-day care of the child, and who is familiar with the individual's developmental history and current behavior. It takes 10 minutes to complete and five minutes to score and was interpreted by the investigators [11]. The p-SRS-2 provides a continuous measure of social ability, with multiple studies demonstrating satisfactory reliability and validity for measuring autism symptoms in pediatric populations [12]. It was completed by the primary caregivers who had knowledge of the individual's abilities across various social settings. It comprises five subscales, i.e., social awareness, social cognition, social communication, social motivation, and restricted interests and repetitive behaviors (RRBs), and takes 15-20 minutes to complete [13].

Statistical analysis

The findings were obtained by using the chi-square test of goodness of fit to assess the demographic data. Continuity correction was used to compare the IQ (BKT), ISAA scores, and T1 Social Responsiveness Scale (SRS) scores with age. As the sample size was 30, tests for normalcy were not deemed necessary. The paired t-test was utilized to compare the mean (SD) of pre-intervention and post-intervention scores of the SCQ and p-SRS-2. Data were analyzed using SPSS Statistics for Windows, version 22.0 (IBM Corp., Armonk, NY), with a level of significance set to p < 0.05.

## Results

Out of the 166 children registered at the Autism Intervention Center, a subset of 30 participants was recruited for the study. All selected children received the allocated intervention and were available for final evaluation, with no participants lost to follow-up. The baseline demographic and socioeconomic factors are detailed in Table 2.

**Table 2:** Baseline demographic and socioeconomic factors * P-value significant at p < 0.05. NIOS: National Institute of Open Schooling.

		N	%	p-value
Gender	Male	26	86.67	<0.0001*
	Female	4	13.33	
Age	8-12 years	15	50	1
	12-15 years	15	50	
School education	Regular school	25	83.33	<0.0001*
	Special school	3	10	
	NIOS	1	3.33	
	Unschooled	1	3.33	
Socioeconomic class (modified Kuppuswamy scale)	Upper	5	16.67	<0.0001*
	Upper middle	22	73.33	
	Lower middle	3	10	
	Upper lower	0	0	
	Lower	0	0	

The mean age of children in the age group of eight to 11 years was 9.49 years. The mean age of children in the age group of 12-15 years was 13.78 years. Age distribution was analyzed with IQ (BKT), ISAA, and pre-therapy (T1) p-SRS2 scores. No significant associations were found between age and IQ-BKT (p = 0.426), age and autism severity (p = 0.06), and age and T1 p-SRS scores (p = 0.145) (Table 3).

**Table 3:** Pre-therapy comparison of IQ (BKT), ISAA, and T1 p-SRS-2 scores with age BKT: Binet Kamat Test of Intelligence; ISAA: Indian Scale for Assessment of Autism; p-SRS-2: Social Responsiveness Scale 2.

		Age group			
	Score range	8-11 years	12-15 years	Total	P-value
BKT	70-90	12	9	21	0.426
	90-110	3	6	9	
	Total	15	15	30	
ISAA	70-108 (Mild)	4	10	14	0.06
	109-153 (Moderate)	11	5	16	
	>153 (Severe)	0	0	0	
	Total	15	15	30	
Pre-therapy p-SRS-2 severity (based on T-score)	60-65 (Mild)	4	1	5	0.145
	66-75 (Moderate)	5	10	15	
	>75 (Severe)	6	4	10	
	Total	15	15	30	

The mean SCQ scores reflected a progressive decline at T1, T2, and T3, indicating a decreasing trend in the severity of the social communication domain. Strong correlation coefficients were observed between pre-therapy (T1) and immediate post-therapy (T2) scores, between pre-therapy (T1) and three months post-therapy (T3) scores, and between immediate post-therapy (T2) and three months post-therapy (T3) SCQ scores. The mean p-SRS-2 scores demonstrate a declining trend at T1, T2, and T3, suggesting a statistically significant decrease in the severity of difficulties in social communication at p < 0.0001. Strong correlation coefficients were observed between T1 and T2, T1 and T3, and T2 and T3 p-SRS-2 scores (Table 4).

**Table 4:** Comparison of T1, T2, and T3 scores of SCQ and p-SRS-2 ^#^ The correlation and t cannot be computed because the standard error of the difference is 0. ** Correlation is significant at the 0.01 level (two-tailed). P-value < 0.05 is significant and p-value < 0.01 is highly significant. SCQ: Social Communication Questionnaire; p-SRS-2: Social Responsiveness Scale 2.

			Mean	Std. deviation	P-value	Correlation coefficient
SCQ T score	Pair 1	T1	21.87	3.371	<0.0001**	0.921
		T2	18.57	3.048		
	Pair 2	T1	21.87	3.371	<0.0001**	0.921
		T3	18.57	3.048		
	Pair 3	T2	18.57^a^	3.048	#	1.000
		T3	18.57^a^	3.048		
p-SRS-2 T score	Pair 1	T1	73.00	6.270	<0.0001**	0.743
		T2	64.57	4.264		
	Pair 2	T1	73.00	6.270	<0.0001**	0.746
		T3	64.30	4.203		
	Pair 3	T2	64.57	4.264	<0.0001**	0.989
		T3	64.30	4.203		

**Figure 2:**
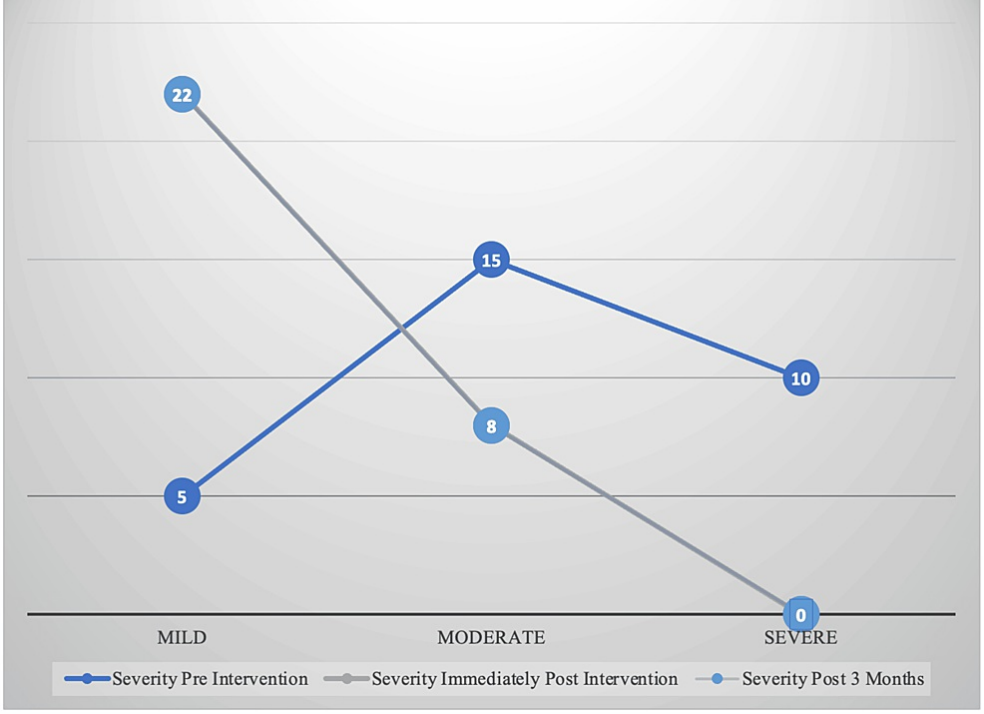
Trend in severity of autism based on p-SRS-2 scores at pre-therapy (T1), immediate post-therapy (T2), and long-term post-therapy (T3) p-SRS-2: Social Responsiveness Scale 2.

Severity based on SRS T-scores was assessed pre-therapy and at T2 and T3. A majority of the 10 initially severe pre-therapy children transitioned to a moderate level of severity, while a smaller portion experienced a decrease in severity to a mild level at T2 and T3. All of the initially moderate pre-therapy children showed a decrease in severity to a mild level. Additionally, the five children who were already mild pre-therapy maintained their mild severity at T2 and T3 (Figure 2).

p-SRS2 subscales were analyzed and significant improvements were observed at T2 and T3 (12 weeks) in total raw and T scores, as well as in all five subscales (awareness, cognition, communication, motivation, and restrictive/repetitive behaviors) compared to pre-therapy values (significant p-values). Benefit sustained in total raw scores, T scores, and social cognition domain from immediate post-therapy to 12 weeks post-therapy in home and school settings (Table 5).

**Table 5:** Comparison of p-SRS-2 subscales at T1, T2, and T3 * P-value is significant at <0.05. ** P-value is highly significant at <0.01.

Variable	Time			P-value		
	T1	T2	T3	T1-T2	T1-T3	T2-T3
Social awareness	13.30 (3.640)	11.20 (3.067)	11.00 (2.828)	<0.0001**	<0.0001**	0.083
Social cognition	16.00 (4.291)	12.00 (3.216)	11.67 (3.273)	<0.0001**	<0.0001**	0.016*
Social communication	36.10 (6.105)	26.33 (5.933)	26.07 (5.783)	<0.0001**	<0.0001**	0.058
Social motivation	15.17 (4.418)	12.07 (3.290)	12.00 (3.414)	<0.0001**	<0.0001**	0.489
Restricted and repetitive behavior	16.77 (4.384)	12.40 (3.529)	12.37 (3.577)	<0.0001**	<0.0001**	0.662
Total raw score	97.63 (18.011)	73.90 (12.452)	72.97 (12.599)	<0.0001**	<0.0001**	0.002**
T score	73.00 (6.270)	64.57 (4.264)	64.30 (4.203)	<0.0001**	<0.0001**	0.030*

There is an improvement in autism severity based on the comparison of pre-therapy SCQ scores with post-therapy immediate and long-term SRS T-scores, supported by statistically significant p-values and strong correlations between the SCQ and SRS scores. Therefore, both scales can be used interchangeably for assessing autism severity (Table 6).

**Table 6:** Correlation of pre-therapy (T1), immediate post-therapy (T2), and long-term (T3) SCQ and p-SRS-2 scores * P-value is significant at <0.05. ** P-value is highly significant at <0.01. ^a ^Correlation coefficient. SCQ: Social Communication Questionnaire; p-SRS-2: Social Responsiveness Scale 2.

	Pre-therapy p-SRS-2 T score (T1)	Immediate post-therapy p-SRS-2 T score (T2)	Long-term p-SRS-2 T score (T3)
Pre-therapy SCQ (T1)	0.215^a^, 0.253 (p-value)	0.461^a^, 0.010 (p-value)*	0.405^a^, 0.027 (p-value)*
Immediate post-therapy SCQ (T2)	0.318^a^, 0.087 (p-value)	0.548^a^, 0.002 (p-value)**	0.514^a^, 0.004 (p-value)**
Long-term SCQ (T3)	0.318^a^, 0.087 (p-value)	0.548^a^, 0.002 (p-value)**	0.514^a^, 0.004 (p-value)**

## Discussion

The pathophysiology of ASD is multifaceted, involving genetic as well as environmental factors. Numerous studies support a strong genetic component in the development of autism, with links to mutations involving specific genes such as SHANK3, NLGN3, and NRXN1 [14]. Studies have observed structural as well as functional differences in the brains of individuals with ASD, with alterations in synaptic connectivity and neurotransmitter dysregulation playing roles in difficulties in mood regulation and social communication skills [15]. Children with ASD often struggle with adapting to the social expectations of society, especially during adolescence [16]. Therapeutic approaches for ASD are diverse, as they involve a multidisciplinary and individualized pattern, due to the variation in needs and strengths of individuals with autism. Some key therapeutic approaches involve applied behavior analysis (ABA), occupational therapy, speech and language therapy, and pharmacological interventions [17,18]. Social skills groups provide an opportunity for individuals with autism to practice social skills in structured environments under the guidance of trained professionals, which can then be applied in community settings. We aimed to assess the impact of GSSI in the age group of eight to 15 years and enhance the transferability of children’s social skills by involving parents so that they can apply the training in the child’s everyday routine. We observed statistically significant improvement in the children's social skills post-intervention.

As the number of high-functioning autism diagnoses among youth continues to rise, our objective was to develop an intervention that was inclusive and tailored to their unique needs and abilities. Our study discovered a consistent decrease in mean SCQ and SRS scores at T1, T2, and T3. The initial improvement in social competence seen after therapy was maintained for 12 weeks across various settings. This suggests that children's social behavior and competence can be sustained without ongoing institutional therapy, with parents playing a crucial role in continuing structured social skills training. This reaffirms the conclusions drawn by Gajre et al. in a similar GSSI among the age group of eight to 12 years [7]. Similar findings were reported in a study by Laugeson et al., showing significant improvements in cooperation, assertion, responsibility, social awareness, social cognition, social communication, social motivation, and reduced autistic mannerisms [19]. Moreover, there were changes in the p-SRS-2 subscale scores of all children between baseline and immediate post-therapy, as well as between baseline and the long-term 12-week follow-up. This aligns with the findings of Corbett et al., who studied the efficacy of peer-mediated intervention on social competence in children with ASD using the SRS [20]. The primary objective of GSSI in ASD is to enhance social skills beyond the training period by involving parents. We draw support from a study conducted by Dekker et al., which demonstrated that involving parents and teachers in GSSI resulted in improvements in social functioning and broad social skills among children with ASD [21]. Additionally, the parents, who were the primary caregivers, served as our informants. Studies exploring the effectiveness of social skills training for individuals with ASD emphasize the importance of intervening during childhood and adolescence. However, few evidence-based interventions specifically target the social competence of teens with high-functioning ASD. This approach promotes the generalization of newly acquired skills. Additionally, the study assesses changes in social competence at a 12-week follow-up, ensuring the maintenance of treatment gains. While the positive outcomes are noteworthy, it is important to acknowledge the presence of limitations in our study. One such limitation is the utilization of parent rating scales as the primary outcome measures. Considering the active involvement of parents in the intervention, there is a possibility of bias. Therefore, incorporating third-party assessments, such as teachers for utilizing the SRS-2 would enhance the validity of the findings. Secondly, conducting further follow-up assessments of social skills at 12 months and 18 months would provide valuable insights into the sustainability of the observed outcomes. Due to the single-arm study design, we encountered challenges in controlling confounding variables and addressing potential placebo effects. Future studies involving a waitlist control group would enable the findings to be generalized to a wider population.

This study aims to provide significant data on the developmental progress of socialization in treated adolescents with ASD. We evaluated the long-term effects of our parent-assisted intervention, considering the inconsistent outcomes reported in previous research regarding the sustainability of results post-conclusion of intervention. Notably, the effectiveness of training varied across different studies for children and adolescents [22-24]. This could result from variations in the implementation of the core methodology, socio-cultural differences among the populations included in different studies, and disparities in age groups and the degree of parental involvement. Nevertheless, our preliminary analyses indicate promising results, suggesting sustained treatment effectiveness over time. Furthermore, the insights gained from this study are likely to contribute to future advancements and improvements in the treatment approach, benefiting both pediatricians and their patients.

## Conclusions

The present study demonstrates an effective therapeutic approach for high-functioning adolescents with ASD, who experience core deficits in social skills, and utilizes a parent-assisted intervention to enhance social skills. Notably, improvements lasted for three months after therapy, highlighting the importance of parent training during sessions. These newly acquired behaviors can positively impact community settings, such as schools and playgrounds, and enhance the overall quality of life for both the child and the caregiver. Our preliminary data can offer insights and serve as a guiding foundation for further research aimed at establishing the efficacy of GSSI as a valuable therapeutic approach.

## References

[1] Faras H, Al Ateeqi N, Tidmarsh L (2010). Autism spectrum disorders. Ann Saudi Med.

[2] Zeidan J, Fombonne E, Scorah J (2022). Global prevalence of autism: a systematic review update. Autism Res.

[3] Laushey KM, Heflin LJ (2000). Enhancing social skills of kindergarten children with autism through the training of multiple peers as tutors. J Autism Dev Disord.

[4] Green SA, Ben-Sasson A (2010). Anxiety disorders and sensory over-responsivity in children with autism spectrum disorders: is there a causal relationship?. J Autism Dev Disord.

[5] Orsmond GI, Krauss MW, Seltzer MM (2004). Peer relationships and social and recreational activities among adolescents and adults with autism. J Autism Dev Disord.

[6] Wolstencroft J, Robinson L, Srinivasan R, Kerry E, Mandy W, Skuse D (2018). A systematic review of group social skills interventions, and meta-analysis of outcomes, for children with high functioning ASD. J Autism Dev Disord.

[7] Gajre MP, Biswas S, Aseri H, Pradhan S (2023). Short-term outcome of social skills group therapy intervention in school aged children with autism. Indian Pediatr.

[8] Roopesh BN (2020). Binet Kamat Test of Intelligence: administration, scoring and interpretation - an in-depth appraisal. Indian J Mental Health.

[9] Mukherjee SB, Malhotra MK, Aneja S, Chakraborty S, Deshpande S (2015). Diagnostic accuracy of Indian Scale for Assessment of Autism (ISAA) in children aged 2-9 years. Indian Pediatr.

[10] Chandler S, Charman T, Baird G (2007). Validation of the social communication questionnaire in a population cohort of children with autism spectrum disorders. J Am Acad Child Adolesc Psychiatry.

[11] Chesnut SR, Wei T, Barnard-Brak L, Richman DM (2017). A meta-analysis of the social communication questionnaire: screening for autism spectrum disorder. Autism.

[12] Chan W, Smith LE, Hong J, Greenberg JS, Mailick MR (2017). Validating the social responsiveness scale for adults with autism. Autism Res.

[13] Bruni TP (2014). Test review: Social Responsiveness Scale-Second Edition (SRS-2). J Psychoeduc Assess.

[14] Boccuto L, Lauri M, Sarasua SM (2013). Prevalence of SHANK3 variants in patients with different subtypes of autism spectrum disorders. Eur J Hum Genet.

[15] Marotta R, Risoleo MC, Messina G, Parisi L, Carotenuto M, Vetri L, Roccella M (2020). The neurochemistry of autism. Brain Sci.

[16] Tantam D (2003). The challenge of adolescents and adults with Asperger syndrome. Child Adolesc Psychiatr Clin N Am.

[17] Foxx RM (2008). Applied behavior analysis treatment of autism: the state of the art. Child Adolesc Psychiatr Clin N Am.

[18] Aman MG (2005). Treatment planning for patients with autism spectrum disorders. J Clin Psychiatry.

[19] Laugeson EA, Frankel F, Gantman A, Dillon AR, Mogil C (2012). Evidence-based social skills training for adolescents with autism spectrum disorders: the UCLA PEERS program. J Autism Dev Disord.

[20] Corbett BA, Key AP, Qualls L, Fecteau S, Newsom C, Coke C, Yoder P (2016). Improvement in social competence using a randomized trial of a theatre intervention for children with autism spectrum disorder. J Autism Dev Disord.

[21] Dekker V, Nauta MH, Mulder EJ, Timmerman ME, de Bildt A (2014). A randomized controlled study of a social skills training for preadolescent children with autism spectrum disorders: generalization of skills by training parents and teachers?. BMC Psychiatry.

[22] Nair MK, Russell PS, George B (2014). CDC Kerala 9: effectiveness of low intensity home based early intervention for autism spectrum disorder in India. Indian J Pediatr.

[23] Pickles A, Le Couteur A, Leadbitter K (2016). Parent-mediated social communication therapy for young children with autism (PACT): long-term follow-up of a randomised controlled trial. Lancet.

[24] Lyra L, Rizzo LE, Sunahara CS, Pachito DV, Latorraca CO, Martimbianco AL, Riera R (2017). What do Cochrane systematic reviews say about interventions for autism spectrum disorders?. Sao Paulo Med J.

